# Effect of dietary sphingomyelin on absorption and fractional synthetic rate of cholesterol and serum lipid profile in humans

**DOI:** 10.1186/1476-511X-12-125

**Published:** 2013-08-19

**Authors:** Vanu R Ramprasath, Peter JH Jones, Donna D Buckley, Laura A Woollett, James E Heubi

**Affiliations:** 1 Richardson Centre for Functional Foods and Nutraceuticals, University of Manitoba, Winnipeg R3T 2N2, Canada; 2 Division of Pediatric Gastroenterology/Hepatology and Nutrition, Children’s Hospital Medical Center, Cincinnati, OH 45229, USA; 3 Department of Pediatrics, Children’s Hospital Medical Center, Cincinnati, OH 45229, USA; 4 Clinical/Translational Research Center, Children’s Hospital Medical Center, Cincinnati, OH 45229, USA; 5 Department of Pathology, Center for Lipid and Atherosclerosis Studies, University of Cincinnati, Cincinnati, OH 45237, USA

**Keywords:** Sphingomyelin, Cholesterol absorption, Fractional synthetic rate, Bile, Human

## Abstract

**Background:**

Diets enriched with sphingolipids may improve blood lipid profiles. Studies in animals have shown reductions in cholesterol absorption and alterations in blood lipids after treatment with sphingomyelin (SM). However, minimal information exists on effect of SM on cholesterol absorption and metabolism in humans. The objective was to assess the effect of SM consumption on serum lipid concentrations and cholesterol metabolism in healthy humans.

**Methods:**

Ten healthy adult males and females completed a randomized crossover study. Subjects consumed controlled diets with or without 1 g/day SM for 14 days separated by at least 4 week washout period. Serum lipid profile and markers of cholesterol metabolism including cholesterol absorption and synthesis were analyzed.

**Results:**

Serum triglycerides, total, LDL- and VLDL- cholesterol were not affected while HDL cholesterol concentrations were increased (p = 0.043) by SM diet consumption. No change in cholesterol absorption and cholesterol fractional synthesis rate was observed with supplementation of SM compared to control. Intraluminal cholesterol solubilization was also not affected by consumption of SM enriched diet.

**Conclusions:**

In humans, 1 g/day of dietary SM does not alter the blood lipid profile except for an increased HDL-cholesterol concentration and has no effect on cholesterol absorption, synthesis and intraluminal solubilization compared to control.

**Trial registration:**

Clinicaltrials.gov # NCT00328211

## Background

Sphingolipids are key components of cell membranes which regulate several functions of the cell [1]. Sphingomyelin (SM), a major part of sphingolipid, is localized to the plasma membrane and lysosomal and golgi membranes [2]. SM is present in considerable quantities in the Western diet [3]. The per capita consumption of SM in the United States is estimated to be 300–400 mg/d; milk (dairy products) and eggs provide 220 mg/d of SM [4,5].

Sphingomyelin plays an important role in cell membrane formation and plasma lipoprotein metabolism including cholesterol efflux, cholesterol absorption, synthesis, and conversion to bile acids, cholesterol esters and other metabolites [5]. ABCG1 mediates the efflux of SM and cholesterol from cell membranes depending on their level and distribution in the membrane [6,7]. Metabolic regulation of SM and cholesterol appear to be inter-coordinated [8,9]. SM has a strong affinity with cholesterol and correlates with the quantity of cholesterol in cell membranes [5,8,10]. As SM binds to cholesterol, it could bind to luminal cholesterol and inhibit absorption directly.

SM may be relatively resistant to solubilization into bile salt micelles or form cholesterol-SM bilayers [11]. SM enriched micelles have been shown to markedly reduce cholesterol solubility compared to phospholipid (PL) enriched micelles [12]. Hence, SM and the ratio of cholesterol to SM have the potential to markedly alter cholesterol trafficking and homeostasis in cells, including enterocytes [4,9]. In addition to affecting sterol balance across cells, SM could affect cholesterol absorption from the lumen of the gut. Indeed, SM has been demonstrated to reduce cholesterol absorption in mice and reduce cholesterol uptake by CaCo2 cells [2,4]. Consumption of SM affects plasma and tissue levels of cholesterol and reduces cholesterol absorption in intestines of rats and mice [13-16]. In mice, diet enriched with 0.1% SM reduced cholesterol absorption by 20.48% [17]. This dose is near the range possible for enrichment of human diets that would allow assessment of the effects of dietary SM on cholesterol absorption and synthesis. Co-administration of cholesterol and SM leads to inhibition of absorption of both lipids in rats [13]. At 1:1 molar ratios (SM/Cholesterol), cholesterol absorption is reduced from 68 to 9% while at ratios of 0.5:1.0, absorption is reduced to 25% [13]. Since the average diet contains 300–400 mg/day of sphingolipid (an amount comparable to dietary cholesterol and accounting for 0.01-0.02% of the diet by weight), it appears very likely that, even without any dietary manipulation, sphingolipids impact cholesterol absorption.

Reduction of LDL cholesterol and elevation of HDL cholesterol by SM consumption in rats and mice makes SM a potential “functional food” [5]. However, Ohlsson et al. found no significant changes in plasma lipid profile after consumption of SM by humans, [18,19]. These studies were free living studies and not well controlled with the background diet during the study. Earlier study [18] was a parallel designed study and the latter one [19] analysed the postprandial lipids with single dose SM administration. Both these studies tested a milk drink -like formulation containing sphingolipids (975 mg) including SM (700 mg), glucoceramides (180 mg) and gangiosides (95 mg). Hence, there is a need of a study with well controlled background diet and cross design to determine the effect of SM when consumed alone on lipid metabolism. There are no such studies with humans to report the effect of SM consumption on cholesterol kinetics including cholesterol absorption and synthesis. The aim of the current study was to analyze the effect of dietary SM supplementation on serum lipids, cholesterol absorption and synthesis and its effects on the intraluminal cholesterol solubilization process under a well controlled situation in humans.

## Results

Ten subjects (5 males and 5 females) of age 32.7 ± 4.1 y; height 170 ± 11 cm; body weight 66.2 ± 9.5 kg and BMI 23 ± 2 kg/m^2^ (Mean ± SD) completed the controlled randomized cross over study. No adverse events were reported during the study. No significant changes in body weights were observed throughout the study.

**Figure 1:**
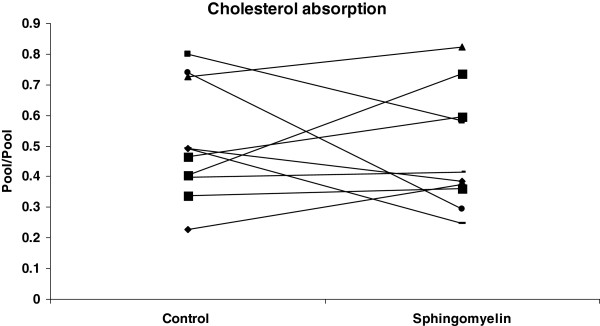
**Effect of SM consumption on cholesterol absorption.** Cholesterol absorption of each subject after consuming a diet with or without SM is expressed as pool/pool. On day 16 of each phase, subjects received oral and intravenous doses of stable isotope labeled cholesterol and blood was collected at baseline and at 24, 48, 72, and 96 hours. Cholesterol absorption was measured using the dual isotope method as described in methods.

**Figure 2:**
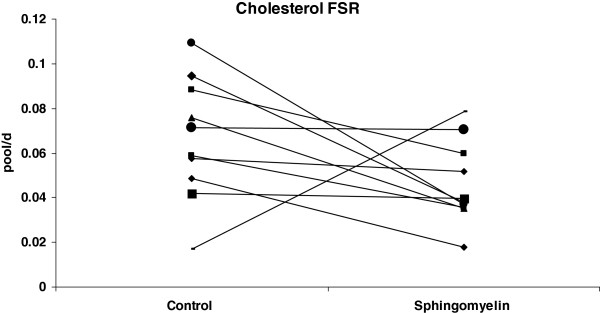
**Effect of SM consumption on cholesterol FSR.** Cholesterol FSR of each subject after consuming a diet with or without SM is expressed as pool/day. On Day 19 of each phase, after an overnight fast, baseline blood was drawn and deuterated water was administered. On day 20, blood was obtained at the same time as isotope administration on Day 19 for measurement of FSR as described in the methods.

Total, LDL- and VLDL-cholesterol in serum were not altered by SM consumption compared with control diet phase (Table 1). Triglyceride concentrations also were unaffected by dietary SM. In contrast, HDL-cholesterol concentrations increased (p = 0.043) with SM supplementation compared to control phase. There was no significant change in cholesterol absorption (Figure 1) or cholesterol FSR (Figure 2) after consumption of diet with SM compared to diet without SM.

**Table 1:** Effect of SM and control diets on serum lipid profile

**Parameter**	**Sphingomyelin diet**	**Control diet**
**Total cholesterol (mg/dL)**	169.90 ± 8.61	155.60 ± 2.99
**Triglycerides (mg/dL)**	81.00 ± 7.63	74.40 ± 10.89
**LDL (mg/dL)**	92.10 ± 4.86	86.20 ± 3.36
**VLDL (mg/dL)**	16.30 ± 1.52	15.78 ± 2.30
**HDL (mg/dL)**	61.50 ± 6.22	54.40 ± 3.85*
**Chol/HDL**	2.91 ± 0.22	2.96 ± 0.22

Values are expressed as mean ± SEM. * indicates significant at p < 0.05 compared with SM diet consumption.

**Figure 3:**
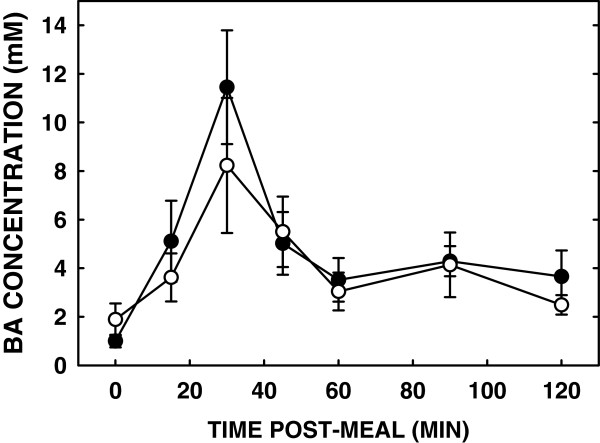
**Effect of SM consumption on luminal bile acid concentrations.** Concentrations of bile acids in the lumen after consumption of a diet with and without SM have been measured and expressed as milli molar concentrations. Open and closed circles represent bile acid concentrations after a SM diet and control diet respectively. Data are presented as means ± SEM.

**Figure 4:**
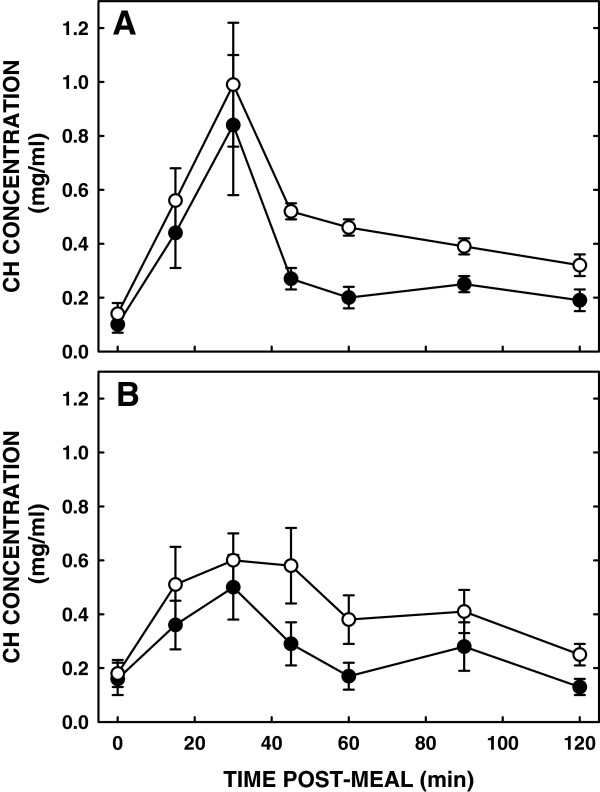
**Effect of SM consumption on luminal total and subphase cholesterol concentrations.** Concentrations of total and subphase cholesterol are shown in panel A and B respectively. Cholesterol content was measured in total luminal content and the aqueous subphase which contains the solubilized cholesterol after ingesting a standardized meal. Open and closed circles represent cholesterol concentrations after a SM diet and control diet respectively. Data are presented as means ± SEM.

**Figure 5:**
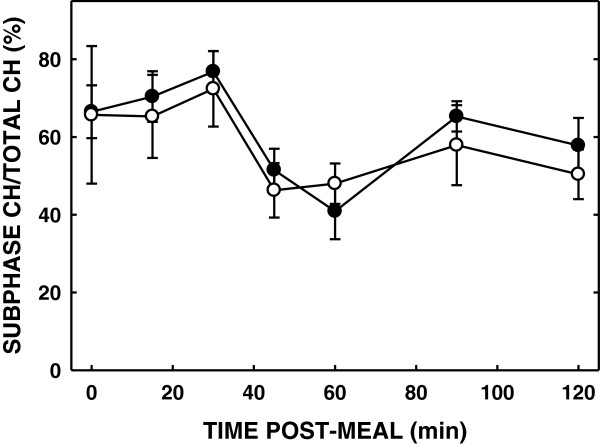
**Effect of SM consumption on ratio of luminal subphase cholesterol concentrations.** Ratios between the total and subphase cholesterol after ingesting a standard meal measured and expressed as percentages. Open and closed circles represent ratios of luminal subphase cholesterol concentrations after a SM diet and control diet respectively. Data are presented as means ± SEM.

Luminal bile acid concentrations were similar after a liquid meal in subjects fed either diet (Figure 3). The amount of micellar (solubilized) cholesterol was similar between the subjects fed SM or control (AUC: 34.2 ± 8.0 vs 39.2 ± 5.6, respectively) (Figure 4). As total cholesterol was also similar, solubilization of cholesterol was the same in subjects fed either diet (Figure 5).

## Discussion

The results of the current study indicate that in humans, dietary SM supplementation of 1 g/day for a diet containing 240 mg cholesterol/day does not affect cholesterol absorption, synthesis or serum lipids except for an effect of raising serum HDL-cholesterol. Current study represents the first definitive study to measure the lipid profile, cholesterol absorption and synthesis in humans under well controlled background diets. Studies in humans, to date, on the effect of SM on cholesterol metabolism or absorption have been limited despite its potential effect demonstrated in rodents and cell culture. Although there has been a human trial with administration of SM enriched formulated drink, study had been conducted under free living conditions without robust control on background diet [18].

Effect of dietary sphingolipids on plasma lipids was examined by Ohlsson et al. [18] in a parallel design of 48 healthy adults who consumed a sphingolipid enriched milk drink containing 975 mg sphingolipid with 700 mg as SM daily for 4 weeks. Consumption of sphingolipids in this amount had no effect on plasma lipids. Previous studies on effect of sphingolipids on postprandial lipid concentrations showed no change in plasma lipid profile. Although our study was only 2 weeks in duration and was 1 g SM/day with a cross over design, we found similar results with the exception of increased HDL-cholesterol with SM dietary enrichment.

Findings from studies with various animal models [13-17] have shown reductions in plasma total and LDL cholesterol along with reduction in intestinal cholesterol absorption. SM supplemented diet (0.5%) for 45 days reduced the hepatic lipids and plasma non HDL cholesterol levels in Zucker fatty rats [16]. Hyperlipidemic APOE*3 Leiden mice consuming a Western diet supplemented with 0.2 and 0.4% sphingolipids for 3 weeks had reduced plasma cholesterol and triglycerides, whereas, lower supplementations (0.1%) had no effect on plasma lipids. When higher levels were fed (1%), cholesterol absorption was reduced by 50% in this animal model leading to a 57 and 58% reduction in plasma total cholesterol and triglycerides respectively [15]. In male Sprague Dawley rats, cholesterol absorption was 19.5% after SM treatment compared to 37.6% in control animals [20].

Rodent studies have also suggested that the ratio between SM and cholesterol may also be a significant determinant for the inhibitory effect of SM on cholesterol absorption. Cholesterol absorption was reduced by different range of molar ratios of SM and cholesterol between 0.5 and 2.6 in rats [13]. Intestinal cholesterol absorption was 17, 9 and 25% when rats treated with SM and cholesterol at 2.6:1, 1:1 and 0.5:1.0 ratio respectively, compared to 68% when treated with cholesterol alone without SM [13]. However, our study used SM to cholesterol molar ratio of 1.0:0.3 which is closer to one of the molar ratios used in the animal study [13] but we found no significant change in either cholesterol absorption or synthesis.

It is disappointing that the previous findings in animal and cell culture studies could not be replicated in humans because of the potential that SM might be used as a functional food. There may be several reasons for these species/experimental condition differences in our findings. SM is not rapidly hydrolysed in intestine in rodents. SMase, which is responsible for digesting SM, is present in very low quantities in pancreatic juice and bile from liver of rats [21]. SMase is an alkaline enzyme that is active at pH 9 and hence is inactive in gastric and duodenal lumen. Hydrolysis of SM in the upper small intestinal lumen is very deliberate, ineffective and incomplete which might be responsible for the inhibitory effect of cholesterol absorption by SM in animal models [22]. In contrast, in humans the hydrolysis of SM is faster and more efficient than rodents as SMase is secreted into the bile [23]. Recently it has been demonstrated that humans can digest and absorb most of the SM consumed in normal diets [24]. It has also been reported that enzymes responsible for digestion of SM including intestinal alkaline SMase and ceramidase are well expressed in humans [25,26]. Unlike animals, SMase is expressed both in the intestine as well as in liver [21,23,27] in humans. However, the digestion of sphingolipid by these enzymes remains poorly defined. The effects of these enzymes may be affected not only by the presence of other lipids but also the ratio between bile salt and SM as well as bile salt and ceramide [28,29].

Although it has been reported that SM could bind to luminal cholesterol and resist solubilization into bile salt micelles [11,12], we found no changes in the concentrations of luminal bile acids and cholesterol in humans in the current study. These findings suggest that the inhibitory effects of SM on cholesterol absorption in rodents is likely different than the effects in humans. Although this is the first definitive study of the effect of SM on cholesterol metabolism and absorption in humans, there were some limitations of current study: 1. The presence of multiple enzymes in the human gut including SMase were not analysed which may explain why we did not see the effects of SM in humans found in rodents. 2. Our sample size was small but given the minimal changes observed a larger sample would likely have shown similar results. 3. There may have been confounding problems with solubilizing SM with olive oil as this may not truly represent how SM might be present in the matrix of a high SM diet; however, this was only practicable way to ensure added SM in the diet. 4. The dose of SM may not have been sufficient to observe changes in cholesterol absorption/metabolism given the proposed higher efficiency of SMase in humans vs. rodents.

The current study included only healthy subjects. More studies may be needed to determine whether SM affects cholesterol absorption and plasma lipids in hyperlipidemic subjects. The dose of SM supplementation used in the current study was 1 g/day and was well tolerated as observed in a previous human study in humans consuming 975 mg per day of sphingolipid or 4 weeks [18]. To determine an inhibitory effect on cholesterol absorption and reduction in plasma lipids, the dose could be increased provided that the increased dose does not cause any deleterious effects. Unfortunately, the cost of such supplementation may make additional supplement prohibitive and identifying foods with high SM content might be a more practicable approach.

In conclusion, consumption of 1 g/day of SM added to the diet in humans does not affect cholesterol absorption, synthesis and blood lipid profile except an increase in HDL. Hence, further studies are needed to investigate the safety and efficacy of SM consumption with optimal higher doses.

## Subjects and Methods

### Human subjects

Healthy adult males and females (n = 10), ages 18–45, were recruited by advertisement. Subjects were screened for cardiovascular, pulmonary, renal, gastrointestinal, hepatobiliary disease or soy allergy and excluded if any exclusionary conditions were found. Subjects with diabetes mellitus and plasma total and LDL cholesterol exceeding 200 mg/dl and 120 mg/dl, respectively, were excluded. Only subjects with Apo A-IV 1/1 and Apo E 3/3 genotypes were enrolled to minimize the effects of variability in cholesterol absorption related to differing genotypes. Women were non-pregnant and were on no oral or implantable contraceptive or took the same contraceptive throughout all sessions of the study.

### Study design and procedures

Once screened and signatures in informed consent forms were obtained, subjects supplied a 3 day diet diary. Based upon caloric intake from the diet diary, the total calories to maintain steady weight were estimated and diet menus prepared on a 3 day rotating schedule. Diets were designed to contain 240 mg cholesterol/day with 30% calories as fat (15% monosaturated) with a P/S ratio of 0.5. Subjects were randomized to diets with or without added 1 g SM, provided as milk SM (Avanti Polar Lipids, Inc. Alabaster AL), dissolved in corn oil and added to the diet at a dose of 1 g/day (Table 2).

**Table 2:** Composition of the background diets

**Composition**	**Quantity**
Proteins (g/100 K calories)	4.95
Carbohydrates (g/100 K calories)	12.58
Fats (g/100 K calories)	3.35
Cholesterol (mg/100 K calories)	11.98
Fatty acids (g/100 g of fat)	
6:0	0.01
8:0	0.39
10:0	0.82
12:0	0.93
14:0	3.04
16:0	19.96
18:0	6.94
20:0	0.33
16:1	1.58
18:1	46.91
20:1	0.20
18:2	16.37
18:3	1.39
SFA (g/100 K calories)	1.00
PUFA (g/100 K calories)	0.49
MUFA (g/100 K calories)	1.31

*P/S* polyunsaturated/saturated fat, *SFA* saturated fatty acids, *PUFA* polyunsaturated fatty acids, *MUFA* monounsaturated fatty acids.

On Day 0, after an overnight fast, subjects visited the General Clinical Research Center (GCRC) at Cincinnati Children’s Hospital Medical Center (CCHMC), Cincinnati, OH, had a physical examination and had blood drawn for serum total, HDL and LDL cholesterol and triglyceride measurement. Thereafter, subjects visited the GCRC every third day to pick up prepared diets which were frozen after preparation. Subjects were weighed weekly to ensure maintenance of basal weight.

After two weeks on the diet (Day 14), study subjects were seen after a 16 hours fast at the GCRC. Urine samples were obtained for pregnancy testing on women of childbearing age. Topical anesthesia was applied to the nose and throat, and sedation was used with intravenous midazolam if subjects wished. A nasoduodenal tube was placed with fluoroscopic guidance with the tube tip at the ligament of Treitz. Subjects ingested a meal consisting of olive oil, egg and egg white, sucrose, vanilla extract and 0.15 M NaCl in 240 ml water [30,31]. Duodenal drainage (15 ml - 20 ml) was collected by siphonage in 15-minute intervals for 1 hour and then 30-minute intervals during the second hour. Aliquots were saved and stored at -70°C for each time period until analysis [32].

On day 16, subjects received oral and intravenous doses of stable isotope labeled cholesterol and blood was collected at baseline and at 24, 48, 72, and 96 hours for measurement of cholesterol absorption using the dual isotope method. On Day 19, cholesterol fractional synthesis rate (FSR) was assessed. After an overnight fast, baseline blood was drawn for RBC cholesterol isotopic ratio and for serum total, HDL, and LDL cholesterol and triglycerides. Subjects were given oral deuterated water and the next day (Day 20), blood was obtained at the same time as isotope administration on Day 19.

After completing the baseline studies, subjects participated in the alternative arm of the study with a washout period of at least 4 week between studies. Thereafter, the same sequence of events was performed as described for the first phase of the study. This study was approved by the Institutional Review Board of CCHMC and the Scientific Advisory Committee of the GCRC.

## Laboratory analyses

### Serum lipid profile analysis

Serum total and HDL cholesterol and triglyceride concentrations were measured enzymatically by Quest Diagnostics Nichols Institute, a certified Center for Disease Control Lipid Research Clinic.

### Cholesterol absorption measurement

Free cholesterol was extracted from RBC according to established methods [33]. ^13^C cholesterol enrichments in RBC lipid extracts were determined using an on-line gas chromatography/combustion/isotope ratio mass spectrometry approach (Agilent 6890 N chromatograph interfaced with a Finnigan Delta V Pulse isotope ratio mass spectrometer (Bremen, Germany)). Isotope abundance, expressed in delta (d) per mil (‰), was calculated using CO_2_ as a reference gas and further corrected against the international reference standard, Pee Dee Belemnite limestone. The measurement of free-cholesterol deuterium enrichment was performed using online gas chromatography/pyrolysis/isotope ratio mass spectrometry and expressed relative to standard mean ocean water and a series of standards of known enrichment. Isotope abundance, expressed in d ‰, was calculated using H_2_ as reference gas. The average ^13^C and D_7_ enrichments of 48 and 72 hours RBC free cholesterol relative to baseline samples were used to calculate the cholesterol absorption coefficient using the ratio of orally ingested ^13^C cholesterol to intravenously administered D_7_ cholesterol as described by Bosner et al. [34] and by us [35].

### Cholesterol FSR determination

Cholesterol synthesis rates were assessed after 24 hours of deuterium water administration using the deuterium incorporation approach [36] as previously described [35]. This method measures cholesterol synthesis as the rate of deuterium incorporation from body water into RBC membrane free cholesterol over a 24 hours period. Deuterium enrichment was measured in both RBC free cholesterol and plasma water as described above. Enrichments were expressed relative to standard mean ocean water using a calibration curve of working standards [36].

### Intraluminal cholesterol solubilization

Duodenal aspirates of 0.5 ml aliquots were saved to represent total luminal contents and 12–15 ml separated into the oil, subphase (aqeous) and pelleted phases by ultracentrifugation at 39,000 rpm for 60 min in a SW40 rotor (Beckman Coulter, Inc., Fullerton, CA) at 37 degrees C as described earlier [32]. Cholesterol was measured in the total and subphase intraluminal contents by gas liquid chromatography; the ratio of subphase to total cholesterol was considered solubilized cholesterol. Bile acid concentrations were measured in the subphase using enzymatic assays (Trinity Biotech, Bray, Ireland).

### Apolipoprotein genotypes

DNA from peripheral blood was isolated according to instructions provided in the High Pure PCR Template Preparation Kit (Boehringer Mannheim, Indianapolis, IN). ApoE and ApoA-IV genotypes were determined as described earlier [37,38].

### Statistical analysis

Results were expressed as mean ± SEM obtained from ANOVA using the root mean square error to estimate the pooled standard error. End point variables were tested for statistical significance by paired *t*-test with p < 0.05. All statistical measures were analyzed using Statistical Package for the Social Sciences version 10.0.

## Abbreviations

SM: Sphingomyelin; GCRC: General clinical research center; CCHMC: Cincinnati children’s hospital medical Center; FSR: Fractional synthesis rate.

## Competing interests

The authors declare that they have no competing interests.

## Authors’ contributions

JEH, LAW and PJ designed research; DDB and JEH coordinated and completed the trial and collected all the data; VRR performed the laboratory analysis, compiled data and performed the statistical analysis; VRR and PJ wrote the final draft and had primary responsibility for the final conduct. All authors read and approved the final manuscript.
